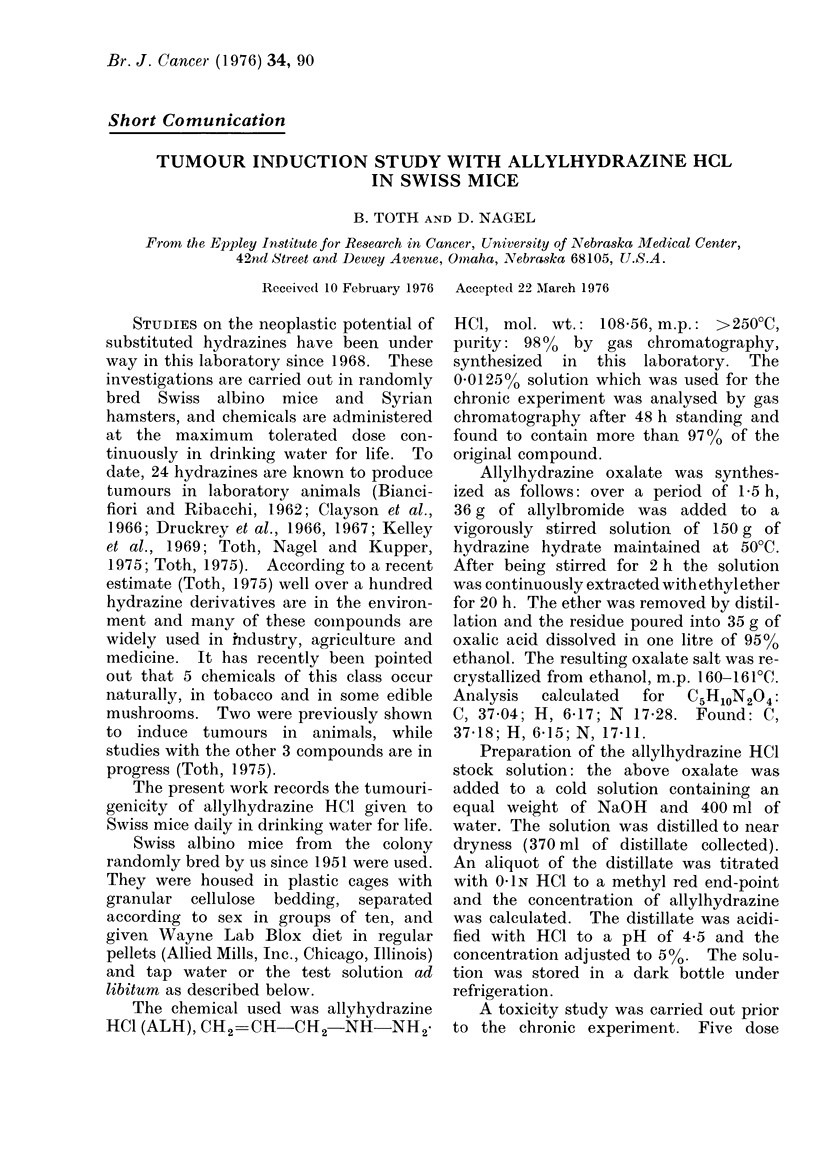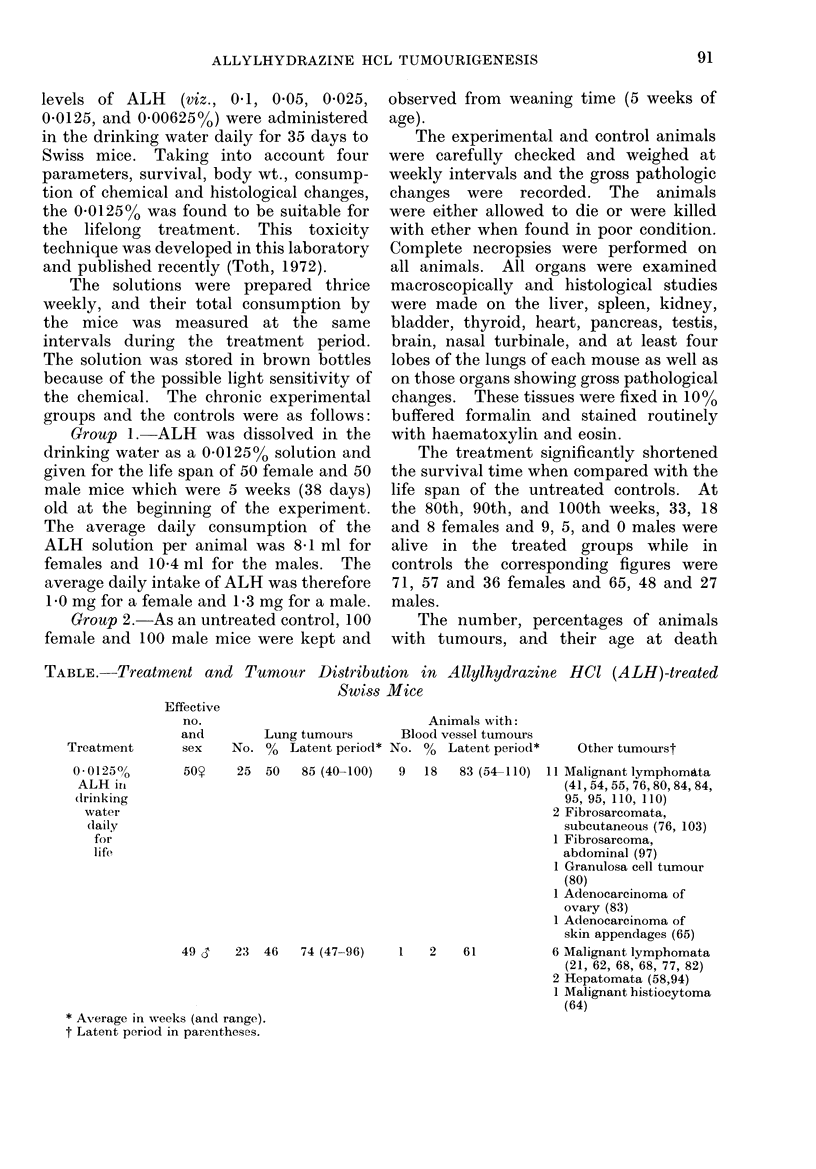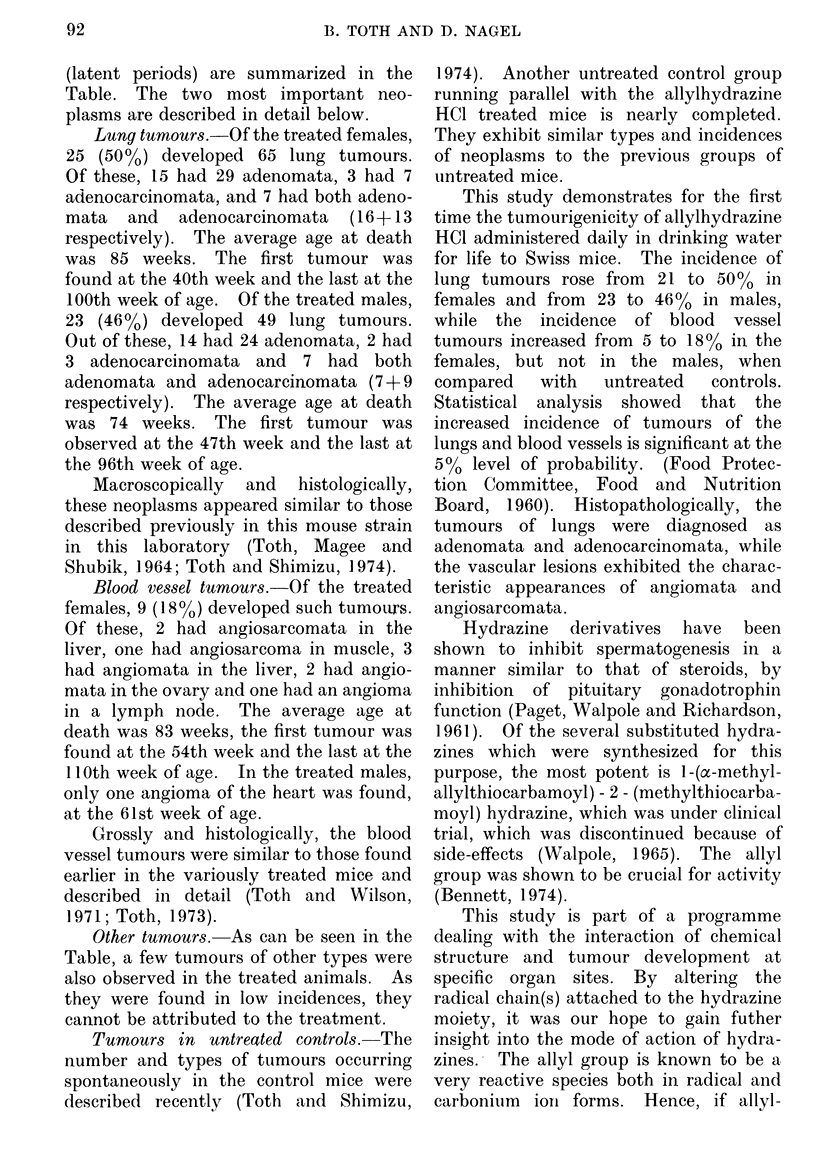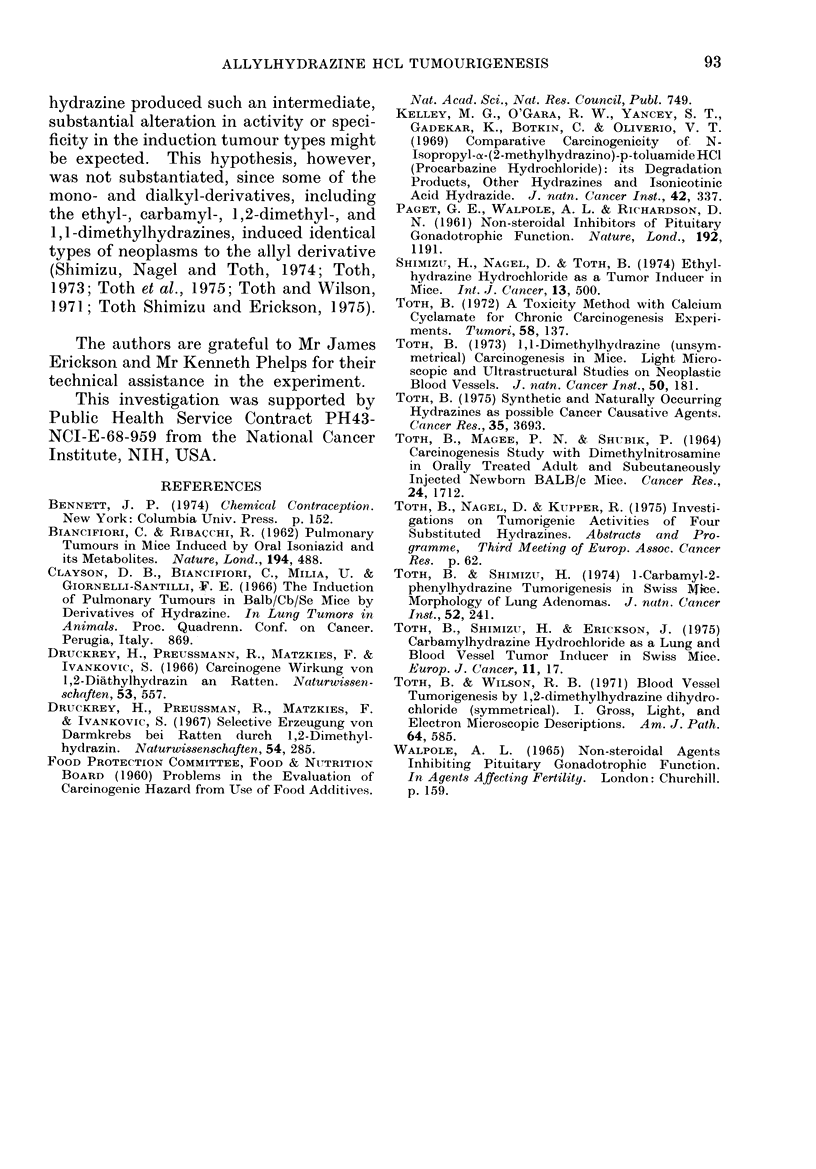# Tumor induction study with allylhydrazine HCl in swiss mice.

**DOI:** 10.1038/bjc.1976.127

**Published:** 1976-07

**Authors:** B. Toth, D. Nagel


*Br. J. Cancer* (1976) **34**, 90

***Short Comunication***

### TUMOUR INDUCTION STUDY WITH ALLYLHYDRAZINE HCL IN SWISS MICE

B. TOTH AND D. NAGEL

*From the Eppley Institute for Research in Cancer, University of Nebraska Medical Center, 42nd Street and Dewey Avenue, Omaha, Nebraska* 68105, *U.S.A.*

Received 10 February 1976 Accepted 22 March 1976

STUDIES on the neoplastic potential of substituted hydrazines have been under way in this laboratory since 1968. These investigations are carried out in randomly bred Swiss albino mice and Syrian hamsters, and chemicals are administered at the maximum tolerated dose continuously in drinking water for life. To date, 24 hydrazines are known to produce tumours in laboratory animals (Biancifiori and Ribacchi, 1962; Clayson *et al.*, 1966; Druckrey *et al.*, 1966, 1967; Kelley *et al.*, 1969; Toth, Nagel and Kupper, 1975; Toth, 1975). According to a recent estimate (Toth, 1975) well over a hundred hydrazine derivatives are in the environment and many of these compounds are widely used in industry, agriculture and medicine. It has recently been pointed out that 5 chemicals of this class occur naturally, in tobacco and in some edible mushrooms. Two were previously shown to induce tumours in animals, while studies with the other 3 compounds are in progress (Toth, 1975).

The present work records the tumourigenicity of allylhydrazine HCl given to Swiss mice daily in drinking water for life.

Swiss albino mice from the colony randomly bred by us since 1951 were used. They were housed in plastic cages with granular cellulose bedding, separated according to sex in groups of ten, and given Wayne Lab Blox diet in regular pellets (Allied Mills, Inc., Chicago, Illinois) and tap water or the test solution *ad libitum* as described below.

The chemical used was allyhydrazine HCl (ALH), $CH_2{=}CH{-}CH_2{-}NH{-}NH_2 \cdot HCl$, mol. wt.: 108·56, m.p.: >250°C, purity: 98% by gas chromatography, synthesized in this laboratory. The 0·0125% solution which was used for the chronic experiment was analysed by gas chromatography after 48 h standing and found to contain more than 97% of the original compound.

Allylhydrazine oxalate was synthesized as follows: over a period of 1·5 h, 36 g of allylbromide was added to a vigorously stirred solution of 150 g of hydrazine hydrate maintained at 50°C. After being stirred for 2 h the solution was continuously extracted with ethyl ether for 20 h. The ether was removed by distillation and the residue poured into 35 g of oxalic acid dissolved in one litre of 95% ethanol. The resulting oxalate salt was recrystallized from ethanol, m.p. 160–161°C. Analysis calculated for $C_5H_{10}N_2O_4$: C, 37·04; H, 6·17; N 17·28. Found: C, 37·18; H, 6·15; N, 17·11.

Preparation of the allylhydrazine HCl stock solution: the above oxalate was added to a cold solution containing an equal weight of NaOH and 400 ml of water. The solution was distilled to near dryness (370 ml of distillate collected). An aliquot of the distillate was titrated with 0·1N HCl to a methyl red end-point and the concentration of allylhydrazine was calculated. The distillate was acidified with HCl to a pH of 4·5 and the concentration adjusted to 5%. The solution was stored in a dark bottle under refrigeration.

A toxicity study was carried out prior to the chronic experiment. Five dose levels of ALH (*viz.*, 0·1, 0·05, 0·025, 0·0125, and 0·00625%) were administered in the drinking water daily for 35 days to Swiss mice. Taking into account four parameters, survival, body wt., consumption of chemical and histological changes, the 0·0125% was found to be suitable for the lifelong treatment. This toxicity technique was developed in this laboratory and published recently (Toth, 1972).

The solutions were prepared thrice weekly, and their total consumption by the mice was measured at the same intervals during the treatment period. The solution was stored in brown bottles because of the possible light sensitivity of the chemical. The chronic experimental groups and the controls were as follows:

*Group* 1.—ALH was dissolved in the drinking water as a 0·0125% solution and given for the life span of 50 female and 50 male mice which were 5 weeks (38 days) old at the beginning of the experiment. The average daily consumption of the ALH solution per animal was 8·1 ml for females and 10·4 ml for the males. The average daily intake of ALH was therefore 1·0 mg for a female and 1·3 mg for a male.

*Group* 2.—As an untreated control, 100 female and 100 male mice were kept and observed from weaning time (5 weeks of age).

The experimental and control animals were carefully checked and weighed at weekly intervals and the gross pathologic changes were recorded. The animals were either allowed to die or were killed with ether when found in poor condition. Complete necropsies were performed on all animals. All organs were examined macroscopically and histological studies were made on the liver, spleen, kidney, bladder, thyroid, heart, pancreas, testis, brain, nasal turbinale, and at least four lobes of the lungs of each mouse as well as on those organs showing gross pathological changes. These tissues were fixed in 10% buffered formalin and stained routinely with haematoxylin and eosin.

The treatment significantly shortened the survival time when compared with the life span of the untreated controls. At the 80th, 90th, and 100th weeks, 33, 18 and 8 females and 9, 5, and 0 males were alive in the treated groups while in controls the corresponding figures were 71, 57 and 36 females and 65, 48 and 27 males.

The number, percentages of animals with tumours, and their age at death

TABLE.—*Treatment and Tumour Distribution in Allylhydrazine HCl (ALH)-treated Swiss Mice*

| | | Animals with: | | | | | | |
|---|---|---|---|---|---|---|---|---|
| | | Lung tumours | | | Blood vessel tumours | | | |
| Treatment | Effective no. and sex | No. | % | Latent period* | No. | % | Latent period* | Other tumours† |
| 0·0125% ALH in drinking water daily for life | 50♀ | 25 | 50 | 85 (40–100) | 9 | 18 | 83 (54–110) | 11 Malignant lymphomata (41, 54, 55, 76, 80, 84, 84, 95, 95, 110, 110)<br>2 Fibrosarcomata, subcutaneous (76, 103)<br>1 Fibrosarcoma, abdominal (97)<br>1 Granulosa cell tumour (80)<br>1 Adenocarcinoma of ovary (83)<br>1 Adenocarcinoma of skin appendages (65) |
| | 49 ♂ | 23 | 46 | 74 (47–96) | 1 | 2 | 61 | 6 Malignant lymphomata (21, 62, 68, 68, 77, 82)<br>2 Hepatomata (58,94)<br>1 Malignant histiocytoma (64) |

* Average in weeks (and range).
† Latent period in parentheses.

(latent periods) are summarized in the Table. The two most important neoplasms are described in detail below.

*Lung tumours*.—Of the treated females, 25 (50%) developed 65 lung tumours. Of these, 15 had 29 adenomata, 3 had 7 adenocarcinomata, and 7 had both adenomata and adenocarcinomata (16+13 respectively). The average age at death was 85 weeks. The first tumour was found at the 40th week and the last at the 100th week of age. Of the treated males, 23 (46%) developed 49 lung tumours. Out of these, 14 had 24 adenomata, 2 had 3 adenocarcinomata and 7 had both adenomata and adenocarcinomata (7+9 respectively). The average age at death was 74 weeks. The first tumour was observed at the 47th week and the last at the 96th week of age.

Macroscopically and histologically, these neoplasms appeared similar to those described previously in this mouse strain in this laboratory (Toth, Magee and Shubik, 1964; Toth and Shimizu, 1974).

*Blood vessel tumours*.—Of the treated females, 9 (18%) developed such tumours. Of these, 2 had angiosarcomata in the liver, one had angiosarcoma in muscle, 3 had angiomata in the liver, 2 had angiomata in the ovary and one had an angioma in a lymph node. The average age at death was 83 weeks, the first tumour was found at the 54th week and the last at the 110th week of age. In the treated males, only one angioma of the heart was found, at the 61st week of age.

Grossly and histologically, the blood vessel tumours were similar to those found earlier in the variously treated mice and described in detail (Toth and Wilson, 1971; Toth, 1973).

*Other tumours*.—As can be seen in the Table, a few tumours of other types were also observed in the treated animals. As they were found in low incidences, they cannot be attributed to the treatment.

*Tumours in untreated controls*.—The number and types of tumours occurring spontaneously in the control mice were described recently (Toth and Shimizu, 1974). Another untreated control group running parallel with the allylhydrazine HCl treated mice is nearly completed. They exhibit similar types and incidences of neoplasms to the previous groups of untreated mice.

This study demonstrates for the first time the tumourigenicity of allylhydrazine HCl administered daily in drinking water for life to Swiss mice. The incidence of lung tumours rose from 21 to 50% in females and from 23 to 46% in males, while the incidence of blood vessel tumours increased from 5 to 18% in the females, but not in the males, when compared with untreated controls. Statistical analysis showed that the increased incidence of tumours of the lungs and blood vessels is significant at the 5% level of probability. (Food Protection Committee, Food and Nutrition Board, 1960). Histopathologically, the tumours of lungs were diagnosed as adenomata and adenocarcinomata, while the vascular lesions exhibited the characteristic appearances of angiomata and angiosarcomata.

Hydrazine derivatives have been shown to inhibit spermatogenesis in a manner similar to that of steroids, by inhibition of pituitary gonadotrophin function (Paget, Walpole and Richardson, 1961). Of the several substituted hydrazines which were synthesized for this purpose, the most potent is 1-(α-methylallylthiocarbamoyl) - 2 - (methylthiocarbamoyl) hydrazine, which was under clinical trial, which was discontinued because of side-effects (Walpole, 1965). The allyl group was shown to be crucial for activity (Bennett, 1974).

This study is part of a programme dealing with the interaction of chemical structure and tumour development at specific organ sites. By altering the radical chain(s) attached to the hydrazine moiety, it was our hope to gain futher insight into the mode of action of hydrazines. The allyl group is known to be a very reactive species both in radical and carbonium ion forms. Hence, if allylhydrazine produced such an intermediate, substantial alteration in activity or specificity in the induction tumour types might be expected. This hypothesis, however, was not substantiated, since some of the mono- and dialkyl-derivatives, including the ethyl-, carbamyl-, 1,2-dimethyl-, and 1,1-dimethylhydrazines, induced identical types of neoplasms to the allyl derivative (Shimizu, Nagel and Toth, 1974; Toth, 1973; Toth *et al.*, 1975; Toth and Wilson, 1971; Toth Shimizu and Erickson, 1975).

The authors are grateful to Mr James Erickson and Mr Kenneth Phelps for their technical assistance in the experiment.

This investigation was supported by Public Health Service Contract PH43-NCI-E-68-959 from the National Cancer Institute, NIH, USA.

### REFERENCES

BENNETT, J. P. (1974) *Chemical Contraception.* New York: Columbia Univ. Press. p. 152.

BIANCIFIORI, C. & RIBACCHI, R. (1962) Pulmonary Tumours in Mice Induced by Oral Isoniazid and its Metabolites. *Nature, Lond.*, **194**, 488.

CLAYSON, D. B., BIANCIFIORI, C., MILIA, U. & GIORNELLI-SANTILLI, F. E. (1966) The Induction of Pulmonary Tumours in Balb/Cb/Se Mice by Derivatives of Hydrazine. *In Lung Tumors in Animals.* Proc. Quadrenn. Conf. on Cancer. Perugia, Italy. 869.

DRUCKREY, H., PREUSSMANN, R., MATZKIES, F. & IVANKOVIC, S. (1966) Carcinogene Wirkung von 1,2-Diäthylhydrazin an Ratten. *Naturwissenschaften*, **53**, 557.

DRUCKREY, H., PREUSSMAN, R., MATZKIES, F. & IVANKOVIC, S. (1967) Selective Erzeugung von Darmkrebs bei Ratten durch 1,2-Dimethylhydrazin. *Naturwissenschaften*, **54**, 285.

FOOD PROTECTION COMMITTEE, FOOD & NUTRITION BOARD (1960) Problems in the Evaluation of Carcinogenic Hazard from Use of Food Additives. *Nat. Acad. Sci., Nat. Res. Council, Publ.* 749.

KELLEY, M. G., O'GARA, R. W., YANCEY, S. T., GADEKAR, K., BOTKIN, C. & OLIVERIO, V. T. (1969) Comparative Carcinogenicity of N-Isopropyl-α-(2-methylhydrazino)-p-toluamide HCl (Procarbazine Hydrochloride): its Degradation Products, Other Hydrazines and Isonicotinic Acid Hydrazide. *J. natn. Cancer Inst.*, **42**, 337.

PAGET, G. E., WALPOLE, A. L. & RICHARDSON, D. N. (1961) Non-steroidal Inhibitors of Pituitary Gonadotrophic Function. *Nature, Lond.*, **192**, 1191.

SHIMIZU, H., NAGEL, D. & TOTH, B. (1974) Ethylhydrazine Hydrochloride as a Tumor Inducer in Mice. *Int. J. Cancer*, **13**, 500.

TOTH, B. (1972) A Toxicity Method with Calcium Cyclamate for Chronic Carcinogenesis Experiments. *Tumori*, **58**, 137.

TOTH, B. (1973) 1,1-Dimethylhydrazine (unsymmetrical) Carcinogenesis in Mice. Light Microscopic and Ultrastructural Studies on Neoplastic Blood Vessels. *J. natn. Cancer Inst.*, **50**, 181.

TOTH, B. (1975) Synthetic and Naturally Occurring Hydrazines as possible Cancer Causative Agents. *Cancer Res.*, **35**, 3693.

TOTH, B., MAGEE, P. N. & SHUBIK, P. (1964) Carcinogenesis Study with Dimethylnitrosamine in Orally Treated Adult and Subcutaneously Injected Newborn BALB/c Mice. *Cancer Res.*, **24**, 1712.

TOTH, B., NAGEL, D. & KUPPER, R. (1975) Investigations on Tumorigenic Activities of Four Substituted Hydrazines. *Abstracts and Programme, Third Meeting of Europ. Assoc. Cancer Res.* p. 62.

TOTH, B. & SHIMIZU, H. (1974) 1-Carbamyl-2-phenylhydrazine Tumorigenesis in Swiss Mice. Morphology of Lung Adenomas. *J. natn. Cancer Inst.*, **52**, 241.

TOTH, B., SHIMIZU, H. & ERICKSON, J. (1975) Carbamylhydrazine Hydrochloride as a Lung and Blood Vessel Tumor Inducer in Swiss Mice. *Europ. J. Cancer*, **11**, 17.

TOTH, B. & WILSON, R. B. (1971) Blood Vessel Tumorigenesis by 1,2-dimethylhydrazine dihydrochloride (symmetrical). I. Gross, Light, and Electron Microscopic Descriptions. *Am. J. Path.* **64**, 585.

WALPOLE, A. L. (1965) Non-steroidal Agents Inhibiting Pituitary Gonadotrophic Function. *In Agents Affecting Fertility.* London: Churchill. p. 159.